# Power Impact of Loop Buffer Schemes for Biomedical Wireless Sensor Nodes

**DOI:** 10.3390/s121115088

**Published:** 2012-11-06

**Authors:** Antonio Artes, Jose L. Ayala, Francky Catthoor

**Affiliations:** 1 Computers Architecture and Automation Department, Complutense University of Madrid, C/ Profesor Jose Garcia Santesmases s/n, 28040 Madrid, Spain; E-Mail: jayala@fdi.ucm.es; 2 Smart Systems and Energy Technology Department, IMEC, Kapeldreef 75, 3001 Leuven, Belgium; E-Mail: catthoor@imec.be

**Keywords:** power impact, loop buffer, instruction memory organisation, biomedical, wireless sensor node

## Abstract

Instruction memory organisations are pointed out as one of the major sources of energy consumption in embedded systems. As these systems are characterised by restrictive resources and a low-energy budget, any enhancement in this component allows not only to decrease the energy consumption but also to have a better distribution of the energy budget throughout the system. Loop buffering is an effective scheme to reduce energy consumption in instruction memory organisations. In this paper, the loop buffer concept is applied in real-life embedded applications that are widely used in biomedical Wireless Sensor Nodes, to show which scheme of loop buffer is more suitable for applications with certain behaviour. Post-layout simulations demonstrate that a trade-off exists between the complexity of the loop buffer architecture and the energy savings of utilising it. Therefore, the use of loop buffer architectures in order to optimise the instruction memory organisation from the energy efficiency point of view should be evaluated carefully, taking into account two factors: (1) the percentage of the execution time of the application that is related to the execution of the loops, and (2) the distribution of the execution time percentage over each one of the loops that form the application.

## 1. Introduction

Embedded systems have different characteristics compared with general-purpose systems. On the one hand, embedded systems combine software and hardware to run a specific and fixed set of applications. However, they differ greatly in their characteristics, because they demand different hardware architectures ranging from multimedia consumer devices to industry control systems. On the other hand, unlike general-purpose systems, embedded systems have restricted resources and a low-energy budget. In addition to these restrictions, embedded systems often have to provide high computation capability, meet real-time constraints, and satisfy varied, tight, and time conflicting constraints in order to make themselves reliable and predictable.

The combination of these requirements, and the fact that the well-known problem of the memory wall becomes even greater in embedded systems, make the decrease of the total energy consumption of the system a big challenge for designers, who not only have to consider the performance of the system but also its energy consumption. Works like [1–3] demonstrate that the Instruction Memory Organisation (IMO) and the Data Memory Hierarchy (DMH) take portions of chip area and energy consumption that are not negligible. In fact, both memory architectures now account for nearly 50%–70% of the total energy budget of an embedded instruction-set processor platform. Therefore, the optimisation in energy consumption of both memory architectures becomes extremely important.

**Figure 1.**
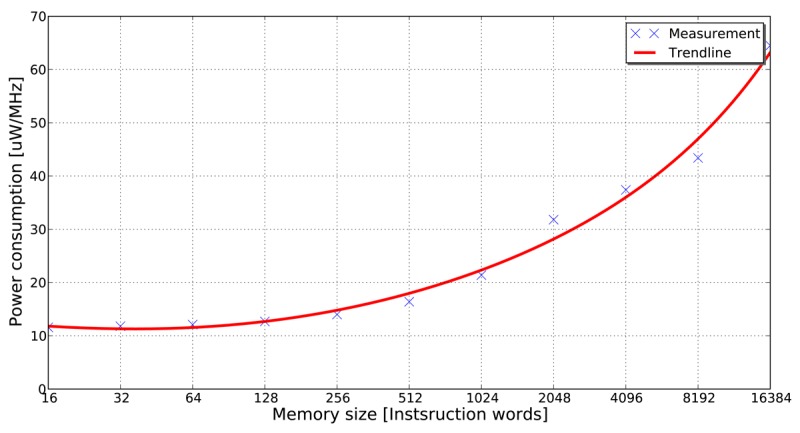
Power consumption per access in 16-bit instruction word SRAM memories designed by Virage Logic Corporation tools [6] using TSMC 90 nm process.

Villarreal *et al.* [4] show that 77% of the total execution time of an application is spent in loops of 32 instructions or less. This fact demonstrates that in applications of signal and image processing, a significant amount of the total execution time is spent in small program segments. If these small program segments can be stored in smaller memory banks (e.g., in the form of loop buffers), the dynamic energy consumption of the system can be reduced significantly. The energy-saving features of the loop buffer concept can be obtained in Figure 1, where it is shown that accesses in a small memory have lower energy consumption than in a large memory. This observation is the base of the loop buffer concept, which is a scheme to reduce the dynamic energy consumption in the IMO. Furthermore, banking is identified as an effective method to reduce the leakage energy consumption in memories [5]. Apart from the possibility of using multiple low-power operating modes, the use of memory banks reduces the effective capacitance as compared with a single monolithic memory.

Embedded systems constitute the digital domain of Wireless Sensor Nodes (WSNs). They are widely deployed in several types of systems ranging from industrial monitoring to medical applications. Particularly, for the biomedical domain, the information that is processed and transmitted is confidential or requires authentication in the majority of the cases. Due to this fact, it is not unusual that two applications like a Heart Beat Detection (HBD) algorithm and a cryptographic algorithm such as Advanced Encryption Standard (AES) algorithm can be found in biomedical WSNs. These two real-life embedded applications are used in this paper as case studies to evaluate the energy reduction achieved by the use of IMOs that are based on the loop buffer concept.

In this paper, the loop buffer concept is applied in the two real-life embedded applications described in the previous paragraph. The loop buffer architectures that are analysed in this paper are the Single Central Loop Buffer and the Banked Central Loop Buffer architecture. The contributions of this paper include:

- An analysis of real-life embedded applications that is used to show which type of loop buffer scheme is more suitable for applications with certain behaviour.
- The use of post-layout simulations to evaluate the power impact of the loop buffer architectures in the experimental evaluation in a strict method in order to have an accurate estimation of parasitics and switching activity.
- Gate-level simulations demonstrate that a trade-off exists between the complexity of the loop buffer architecture and the power benefits of utilising it. The use of loop buffer architectures in order to optimise the IMO from the energy efficiency point of view should be evaluated carefully. Two factors have to be taken into account in order to implement an energy efficient IMO based on a loop buffer architecture: (1) the percentage of the execution time of the application that is related to the execution of the loops included in the application, and (2) the distribution of the execution time percentage, which is related to the execution of the loops, over each one of the loops that form the application.

The rest of the paper is organised as follows. Section 2 presents the related work regarding the loop buffer concept. Section 3 describes the applications that are used as case studies, while Section 4 describes the experimental framework. Section 5 shows and analyses the results of the simulations. Finally, in Section 6 the conclusions are presented.

## 2. Related Work

During the last 10 years, researchers have demonstrated that the IMO can contribute to a large percentage of the total energy consumption of the system (e.g., [3]). Most of the architectural enhancements that have been used to reduce this energy consumption have made use of the loop buffer concept. Works [7–15] present the most traditional use of the loop buffer concept: the Single Central Loop Buffer (SCLB) architecture. Work [7] analysed three hardware techniques to improve direct-mapped cache performance: miss caching, victim caching, and stream buffers prefetch. Work [8] proposed a configurable instruction cache, which could be tailored for a particular application in order to utilise the sets efficiently, without any increase in the cache size, associativity, or cache access time. Work [9] proposed an alternative approach to detect and remove unnecessary tag-checks at run-time. Using execution footprints, which were recorded previously in a branch target buffer, it was possible to omit the tag-checks for all instructions in a fetched block. If loops could be identified, fetched, and decoded only once, work [10] proposed an architectural enhancement that could switch off the fetch and decode logic. The instructions of the loop were decoded and stored locally, from where they were executed. The energy savings came from the reduction in memory accesses as well as the lesser use of the decode logic. In order to avoid any performance degradation, work [11] implemented a small instruction buffer that was based on the definition, detection and utilisation of special branch instructions. This architectural enhancement had neither an address tag store nor valid bit associated with each loop cache entry. Work [12] evaluated the Filter Cache. This enhancement was an unusually small first-level cache that sacrificed a portion of performance in order to save energy. The program memory was only required when a miss occurs in the Filter Cache, otherwise it remained in standby mode. Based on this special loop buffer enhancement, work [13] presented an architectural enhancement that detected the opportunity to use the Filter Cache, and enabled or disabled it dynamically. Also, work [14] introduced a Decoder Filter Cache in the IMO to provide directly decoded instructions to the processor, reducing the use of the instruction fetch and decode logic. Furthermore, work [15] proposed a scheme, where the compiler generated code in order to reduce the possibility of a miss in the loop buffer cache. However, the drawback of this work was the trade-off between the performance degradation and the power savings, which was created by the selection of the basic blocks.

Parallelism is a well-known solution for increasing performance efficiency. Because loops form the most important part of an application [4], loop transformation techniques are applied to exploit parallelism within loops on single-threaded architectures. Centralised resources and global communication make these architectures less energy efficient. In order to reduce these bottlenecks, several solutions that used multiple loop buffers were proposed in literature. Works [16–18] are examples of the work done in this field: the Multiple Central Loop Buffer (MCLB) architecture. On the one hand, work [16] presented a distributed control-path architecture for Distributed Very Long Instruction Word (DVLIW) processors, which overcame the scalability problem of Very Long Instruction Word (VLIW) control-paths. The main idea was to distribute the fetch and decode logic in the same way that the register file was distributed in a multi-cluster data-path. On the other hand, work [17] proposed a multi-core architecture that extended traditional multi-core systems in two ways. First, it provided a dual-mode scalar operand network to enable efficient inter-core communication without using the memory. Second, it could organise the cores for execution in either coupled or decoupled mode through the compiler. In coupled mode, the cores executed multiple instructions streams in lock-step to collectively work as a wide-issue VLIW. In decoupled mode, the cores executed independently a set of fine-grain communicating threads extracted by the compiler. These two modes created a trade-off between communication latency and flexibility, which should be optimised depending on the required parallelism. Work [18] analysed a set of architectures for efficient delivery of VLIW instructions. A baseline cache implementation was compared with a variety of organisations, where the evaluation included the cost of the memory accesses and the wires that were necessary to distribute the instruction bits.

The SCLB architecture and the MCLB architecture can be implemented based on memory banks or without them. Power management of banked memories has been investigated from different angles including hardware, OS and compiler. Using memory access patterns in embedded systems, Benini *et al.* [19] proposed an algorithm to partition on-chip SRAM into multi-banks that could be accessed independently. Fan *et al.* [20] presented memory controller policies for memory architectures with low-power operating modes. Lyuh *et al.* [21] used a compiler directed approach to determine the operating modes of memory banks after scheduling the memory operations. As we can see from previous approaches, the drawback of using multiple buffers is usually to increase the logic that controls the banks, which has the benefit of further decreasing the leakage energy consumption. This fact leads to the increase of the interconnect capacitances, as well as the reduction of possible dynamic energy savings that are related to the access to smaller memories. Most approaches that are related to caches assume that automated tuning is done statically, meaning that the tuning is done once during application design time. Ghosh *et al.* [22] presented a heuristic that, through an analytical model, directly determined, based on the designer's performance constraints and application characteristics, the configuration of the cache. Other cache tuning approaches could be used dynamically, while an application was executed [23].

In this paper, an experimental framework is developed in order to evaluate the SCLB architecture and the Banked Central Loop Buffer (BCLB) architecture from energy consumption point of view.

## 3. Experimental Applications

Two real-life embedded applications that can be found in biomedical WSNs are used as case studies in this paper. Both applications are described in the following Subsections.

### 3.1. HBD Algorithm

**Figure 2.**
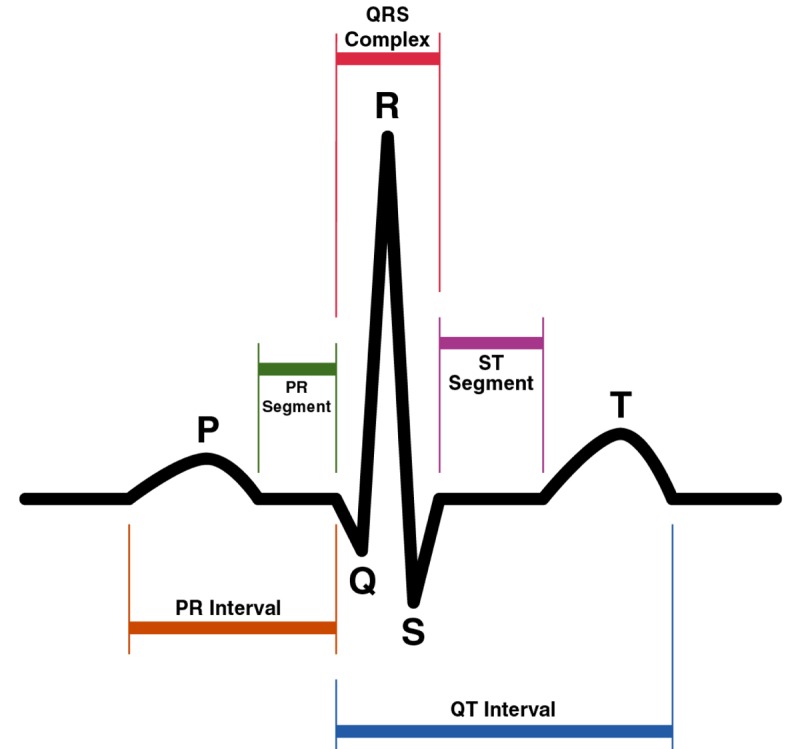
P, Q, R, S and T waves on an ECG signal.

The Heart Beat Detection (HBD) algorithm is a biomedical application based on a previous algorithm that was developed by [24]. This algorithm uses the Continuous Wavelet Transform (CWT) [25] to detect heartbeats automatically. The QRS complex is the part of an Electrocardiogram (ECG) signal that represents the greatest deflection from the baseline of the signal, and is where this algorithm tries to detect the R-peak. Figure 2 shows the P, Q, R, S, and T waves on an ECG signal.

**Figure 3.**
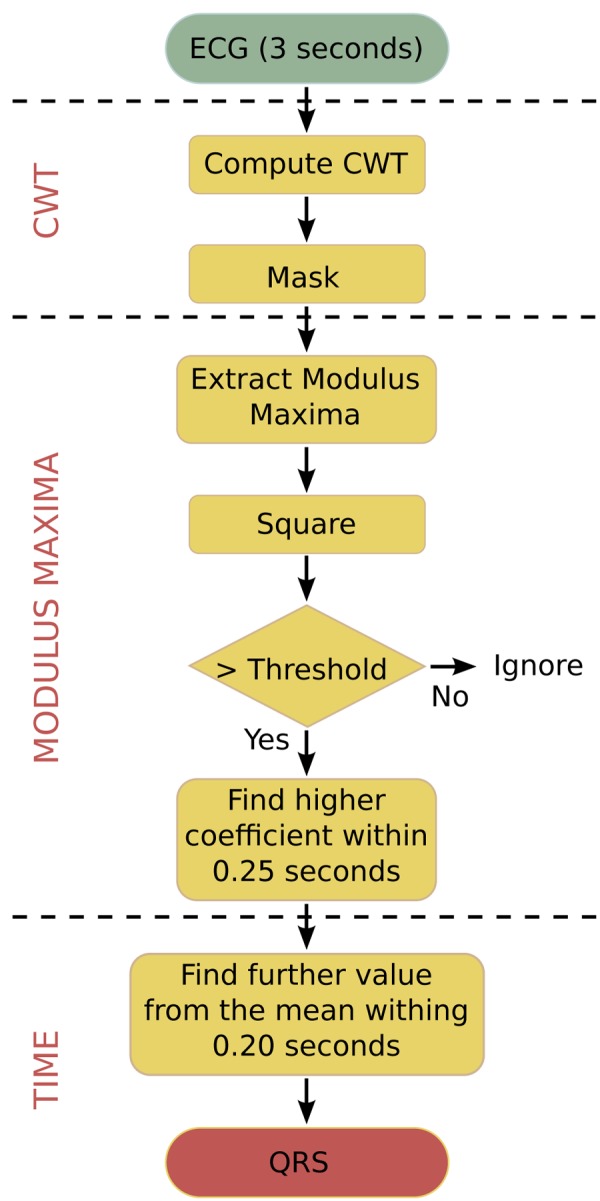
Flowchart of the HBD algorithm.

The algorithm that is used in this paper is an optimised C-language version for biomedical WSNs. It does not require pre-filtering and is robust against interfering signals under ambulatory monitoring conditions. The algorithm works with an input frame of 3 seconds, which includes 2 overlaps of 0.5 seconds between consecutive frames in order to not lose data between frames. Figure 3 shows the flowchart of this algorithm. The algorithm performs the following steps to process an input data frame:

1. The ECG signal is analysed within a window of 3 seconds, where the CWT is calculated over this interval and a mask is applied to remove edge components.
2. The square of the modulus maxima of the CWT is taken in order to emphasise the differences between coefficients. Values above a chosen threshold are selected as possible R-peaks.
3. In order to separate the different peaks, all modulus maxima points within intervals of 0.25 seconds are analysed in turn as search intervals. In every search interval, the point with the maximum coefficient value is selected as R-peak.
4. The algorithm finds the exact location of the R-peak in the time-domain.

The input of this algorithm is an ECG signal from MIT/BIH database [26]. The output is the positions in time-domain of the heartbeats that are included in the input frame. The testing of this optimised algorithm results in a sensitivity of 99.68% and a positive predictivity of 99.75% on the MIT/BIH database.

### 3.2. AES Algorithm

The Advanced Encryption Standard (AES) algorithm is a cryptographic application. The algorithm used in this paper is the security operation mode AES-CCM-32. This mode of operation provides confidentiality, data integrity, data authentication, and replay protection. In the next paragraphs, AES and CCM are explained.

AES [27] is a symmetric-key encryption standard in which both the sender and the receiver use a single key for encryption and decryption. The data block length that is used by this algorithm is fixed to 128 bits, while the length of the cipher key can be 128, 192 or 256 bits. The AES algorithm is an iterative algorithm in which the iterations are called rounds, and the total number of rounds can be 10, 12 or 14, depending on whether the key length is 128, 192, or 256 bits, respectively. The data block that is processed during the rounds is called State. In the encryption process, each round, except for the final round, consists of four transformations:

- **SubBytes** is a byte substitution transformation that can be implemented in software in two ways: based on finite fields digital logic or as a look-up table (S-Box lut).
- **ShiftRows** shifts cyclically the rows of the State, over a different number of bytes.
- **MixColumns** multiplies the columns of the State with a fixed polynomial.
- **AddRoundKey** applies a XOR operation between the State and a Round key.

**Figure 4.**
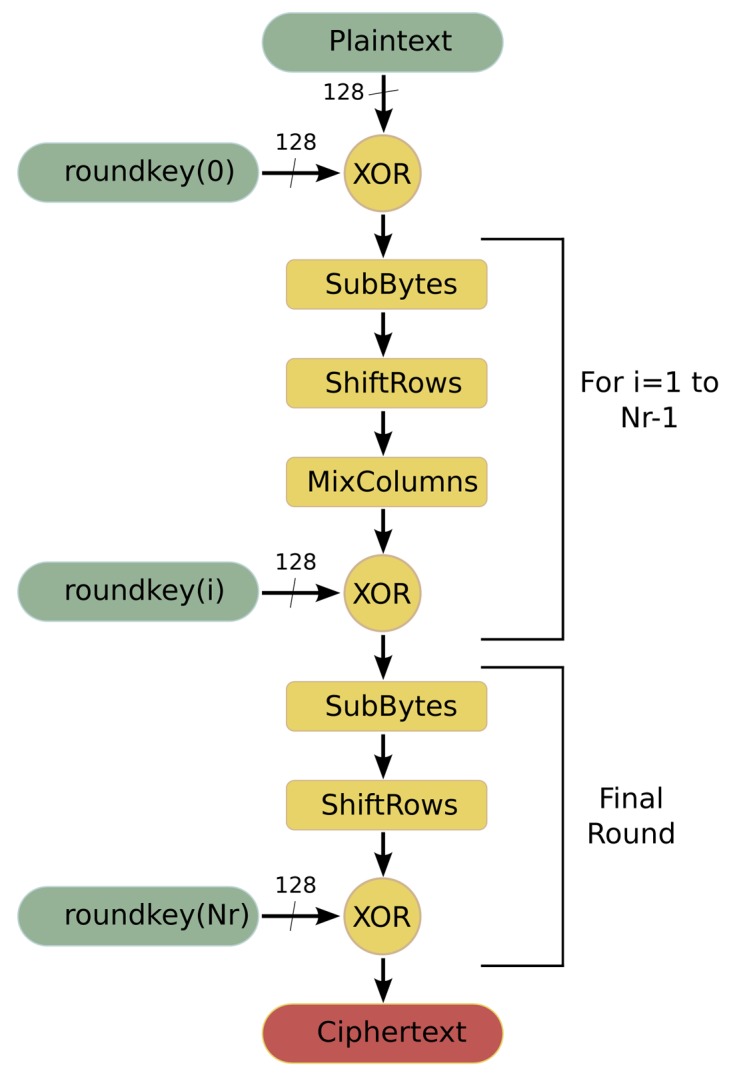
Flowchart of the AES algorithm. Encryption process.

The final round does not have the MixColumns transformation. Figure 4 shows the flowchart of this algorithm when it is working in encryption mode.

The CCM (CTR-CBC-MAC), which is presented in the NIST Special Publication 800-38C [28], encrypts and authenticates the message and the associated data. Depending on the size of the message authentication code that it produces (4, 8, or 16 bytes), three different variations of AES-CCM exist: AES-CCM-32, AES-CCM-64, and AES-CCM-128.

Because biomedical WSNs have ultra-low power requirements, the proposed algorithm supports only 128-bit key. In addition, only the encryption mode of the AES algorithm is supported. However, with a very small change in the design, both encryption and decryption can be supported. In this algorithm, the input data frame is fixed to 1,460 bytes of information, whereas the output is a data packet where the information is encrypted.

## 4. Experimental Framework

This Section describes all the components of the system that form the experimental framework. On the one hand, Subsections 4.1, 4.2, and 4.3 describe the processor architectures that are used in this paper. On the other hand, Subsection 4.4 presents the rest of the components that form the experimental framework and explains how the experimental framework is built based on a platform that can contain any processor.

### 4.1. General-Purpose Processor

The general-purpose processor architecture is designed using the tools from Target Compiler Technologies [29]. The Instruction-Set Architecture (ISA) of this processor is composed of integer arithmetic, bitwise logical, compare, shift, control, and indirect addressing I/O instructions. Apart from support for interrupts and on-chip debugging, this processor supports zero-overhead looping control hardware, which allows fast looping over a block of instructions. Once the loop is set using a special instruction, additional instructions are not needed in order to control the loop, because the loop is executed a pre-specified number of iterations (known at compile time). This loop buffer implementation supports branches, and in cases where the compiler cannot derive the loop count, it is possible to inform the compiler through source code annotations that the corresponding loop will be executed at least N times, and at most M times, such that no initial test is needed to check whether the loop has to be skipped. The special instruction that controls the loops introduces only one cycle delay. The status of this dedicated hardware is stored in the following set of special registers:

**Loop Start address register (LS)** It stores the address of the first instruction of the loop.
**Loop End address register (LE)** It stores the address of the last instruction of the loop.
**Loop Count register (LC)** It stores the remaining number of loop iterations.
**Loop Flag register (LF)** It keeps track of the hardware loop activity. Its value represents the number of nested loops that are active.

The experimental framework uses an I/O interface to provide the capability of receiving and sending data in real-time. This interface is implemented in the processor architecture by FIFOs that are directly connected to the register file. The data memory that is required by this processor architecture in order to be a general-purpose processor is a memory with a capacity of 16k words/16 bits, whereas the required program memory is a memory with a capacity of 2 k words/16 bits.

**Figure 5.**
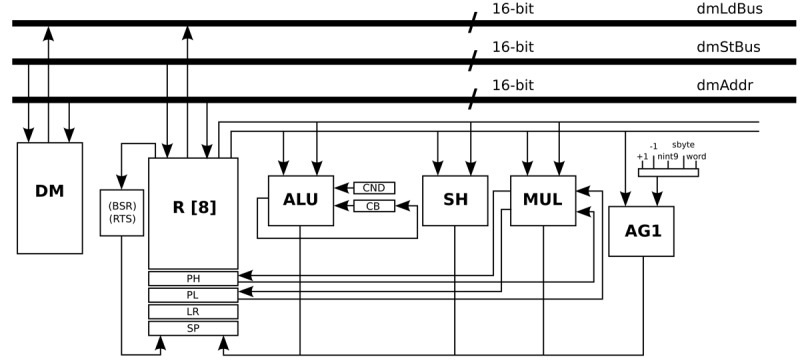
Data-path of the general-purpose processor.

Figure 5 presents the data-path of this processor, where the main blocks are Data Memory (DM), Register File (R), Arithmetic Logic Unit (ALU), Shift Unit (SH), Multiplication Unit (MUL), and Address Generation unit (AG1). The address generation unit specifies the next address as normal word instruction in the case of word label, as negative offsets to the stack pointer register in the case of the nint9 label, and as relative offset of short jump instructions in the case of the sbyte label.

## 4.2. Optimised Processor for the HBD Algorithm

The processor that is optimised for the HBD algorithm is based on the processor architecture that is presented in Subsection 4.1. This Subsection presents the modifications and optimisations that are performed in order to build this optimised processor.

From the deep analysis that has to be performed to design the Application-Specific Instruction-set Processor (ASIP) for the HBD algorithm, a loop is pointed out as the performance bottleneck in this specific algorithm. This loop performs the convolution operation, which is the core of the CWT. A signed multiplication, whose result is accumulated in a temporally variable, is performed inside of this critical loop. The execution of this instruction is 72% of the execution time of the algorithm according to profiling information. Therefore, in order to improve the performance, the MUL unit is modified to multiply two signed integers and accumulate, without shifting, the result of the multiplication. This optimisation saves energy and at the same time reduces both the complexity of the MUL unit and the execution time of the application.

The load operations that are related with the previous MUL operation are combined in a customised instruction in order to be executed in parallel. However, in the general-purpose processor, it is only possible to load and store data from the same memory once per stage of the pipeline. To solve this bottleneck, the main data memory is split in two identical data memories: Data Memory (DM) and Constant Memory (CM). In order to access two memories in parallel, another address generator (AG2) is created such that the load and store operations from the DM and CM can be performed at the same stage of the pipeline.

As the input registers of the MUL unit can be loaded directly, a new modification can be performed. The parallel load and MUL instruction are combined, by adding another stage in the pipeline and creating a custom instruction that integrates both instructions. The MUL instruction is then executed in the second stage of the pipeline, while the parallel load instruction is executed in the first stage of the pipeline. After this last modification, the MUL operation that is included in the main critical loop of this algorithm is performed using only one assembly instruction.

In a similar way as the MUL operation, another critical loop is optimised by combining load, select, and equal instructions in order to be executed in parallel. This instruction is created adding the functionality of the equal and select instructions, and combining both of them with a normal load operation. The functional unit ALU 2 is created for this specific operation.

It is necessary to remark that, apart from the specialised instructions that are described in previous paragraphs, custom techniques like source code transformations (e.g., function combination, loop unrolling) and mapping optimisations (e.g., use of look-up tables, elimination of divisions and multiplications, instruction set extensions) are applied to generate a more efficient code.

**Figure 6.**
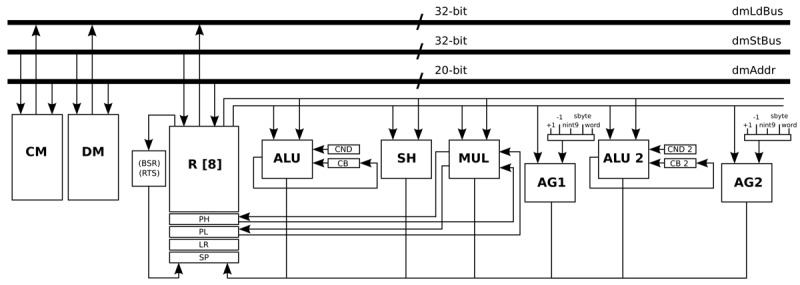
Data-path of the processor that is optimised for the HBD algorithm.

All the optimisations and modifications that are described in this Subsection result in a new processor architecture shown in Figure 6. Basically, an address generator (AG2) and a second ALU (ALU 2) are added, in addition to some pipes and ports. Apart from that, the Program Counter (PC) is modified to handle instruction words that use 32-bit immediate values. In order to handle ECG signals sampled at 1,000 Hz, the memories that are required by this processor architecture are a DM with a capacity of 8 k words/32 bits, and a CM with a capacity of 8 k words/32 bits. Besides, the program memory that is required by this processor architecture is a memory with a capacity of 1 k words/20 bits. This optimised processor is an implementation that is based on the work presented in Reference [30].

## 4.3. Optimised Processor for the AES Algorithm

The processor that is optimised for the AES algorithm is based also on the processor architecture that is presented in Subsection 4.1. This Subsection presents the modifications and optimisations that are performed in order to build this optimised processor.

Analysing this algorithm, the critical functions are identified and optimised in order to improve performance in terms of clock cycles and memory accesses. Custom techniques like source code transformations (e.g., function combination, loop unrolling) and mapping optimisations (e.g., use of look-up tables, elimination of divisions and multiplications, instruction set extensions) are applied to generate a more efficient code.

In the design of this optimised processor, the structure of the general-purpose architecture is kept intact (16-bit data-path), and an extra 128-bit data-path is added. This last data-path is connected with a vector memory, a vector register file, and a vector unit. The vector unit includes the AES accelerating operations, as well as the logic and arithmetic instructions that this algorithm requires. In this processor, the ISA was also extended with one AES accelerating instruction that has two inputs: a 128-bit input, which can be the State or a Round key, and an integer input, which indicates the behaviour of the instruction itself. Depending on the input, the output contains the State or a Round key. One of the advantages of this design is the ability to use the larger vector units only when they are required.

**Figure 7.**
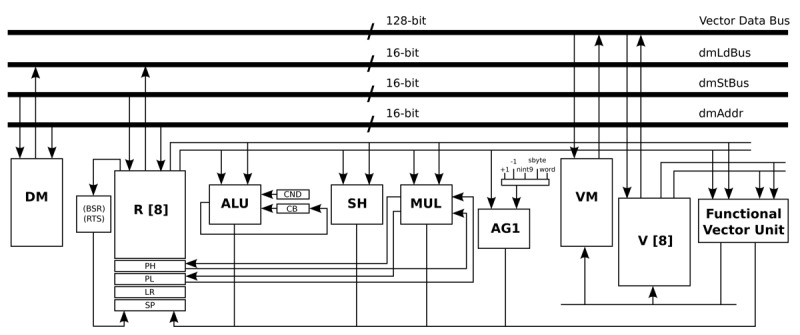
Data-path of the processor that is optimised for the AES algorithm.

All the optimisations and modifications that are presented in this Subsection result in the new processor architecture shown in Figure 7. Basically, an extra 128-bit data-path is added. This extra data-path includes a Vector Memory (VM), a Vector register file (V), and a Vector Unit (Functional Vector Unit). In order to handle an input signal of 1,460 bytes, the data memory required by this processor architecture is a memory with a capacity of 1 k words/16 bits, and the VM is a memory with a capacity of 64 words/128 bits. On the other hand, the required program memory is a memory with a capacity of 1 k words/16 bits. This optimised processor is an implementation that is based on the work presented in [31].

## 4.4. Experimental Platform

The experimental platform is automatically generated for any of the processors described in Subsections 4.1, 4.2, and 4.3. The experimental platform is composed of a DMH, an IMO, an I/O interface, and a processor that is used as core of the platform. On the one hand, the program memory and the data memory are SRAM memories designed by Virage Logic Corporation tools [6]. On the other hand, the I/O interface that provides the capability to receive and send data in real-time is connected with the I/O interface that is described in Subsection 4.1.

**Figure 8.**
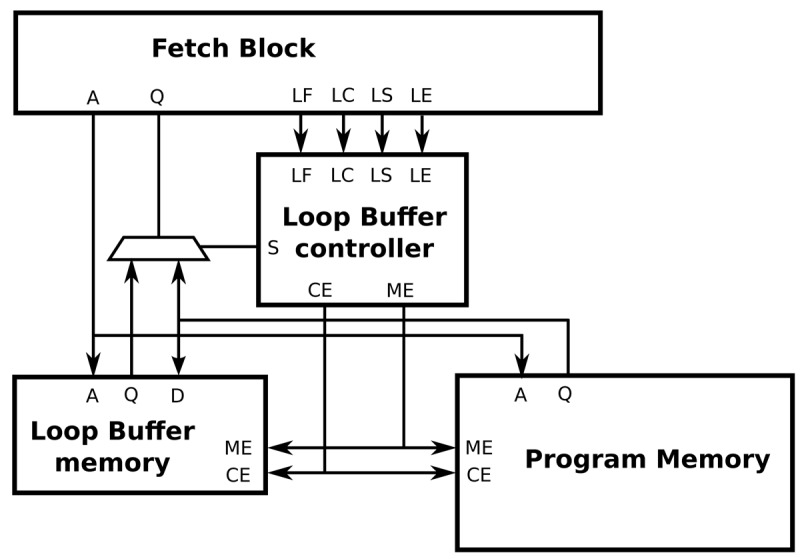
IMO interface for a SCLB architecture.

The interface between a processor architecture and an IMO is depicted in Figure 8. The interconnections of the processor architecture, the program memory, the loop buffer memory and the loop buffer controller are included in this figure. Every component that forms the IMO is explained in the next paragraphs. In our experimental platform, the loop buffer architecture, which is composed of the loop buffer memory and the loop buffer controller, can be configurable to be used as an SCLB or BCLB architecture. For simplicity, the SCLB architecture is used in the next paragraphs to explain the loop buffer concept operation.

In essence, the operation of the loop buffer concept is as follows. During the first iteration of the loop, the instructions are fetched from the program memory to both loop buffer architecture and processor. In this iteration, the loop buffer architecture records the instructions of the loop. Once the loop is stored, for the rest of iterations, the instructions are fetched from the loop buffer architecture instead of the program memory. In the last iteration, the connection between the processor and program memory is restored, such that subsequent instructions are fetched from the program memory. During the execution of non-loop parts of the application code, instructions are fetched directly from the program memory.

**Figure 9.**
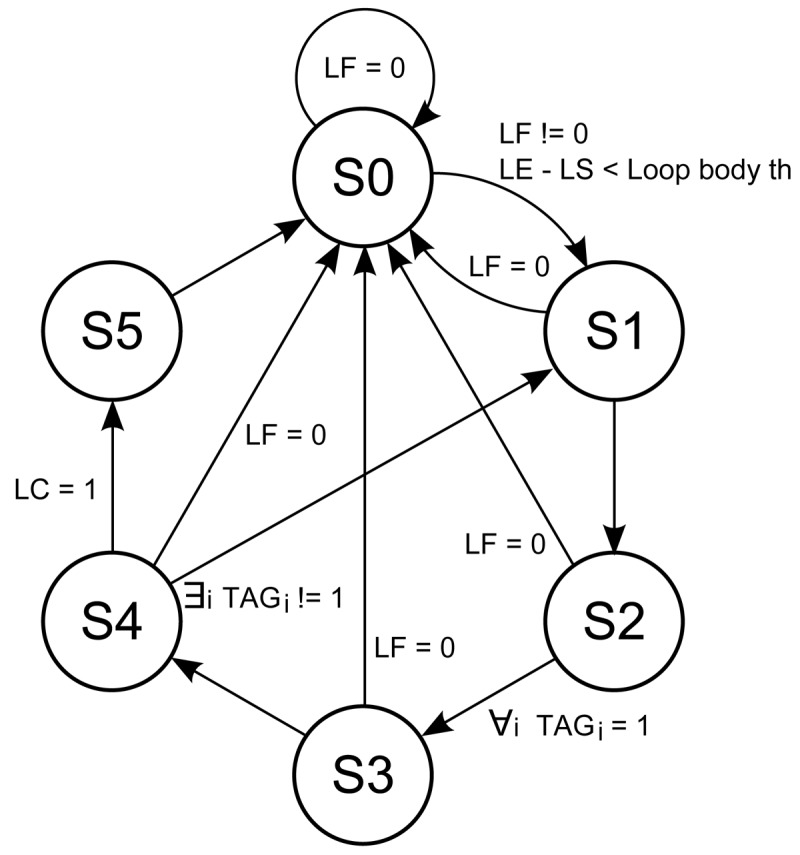
State-machine diagram of the loop buffer controller.

The loop buffer controller monitors the operation of the loop buffer architecture based on a state-machine. This state-machine is shown in Figure 9. The six states of the state-machine are:

**s0** Initial state.
**s1** Transition state between *s0* and *s2*.
**s2** State where the loop buffer is recording the instructions that the program memory supplies to the processor.
**s3** Transition state between *s2* and *s4*.
**s4** State where the loop buffer is providing the instructions to the processor.
**s5** Transition state between *s4* and *s0*.

The transition states *s1*, *s3*, and *s5* are necessary in order to give the control of the instruction supply from the program memory to the loop buffer architecture and vice-versa. The transition between *s4* and *s1* is necessary because the body size of a loop can change in real-time (*i.e.*, in a loop body with if-statements or function calls). In order to check in real-time whether the loop body size changes or not, a 1-bit tag is used. This tag is associated with each address that is stored in the loop buffer. The loop buffer controller checks this tag to know if the address is already stored in the loop buffer or not.

**Figure 10.**
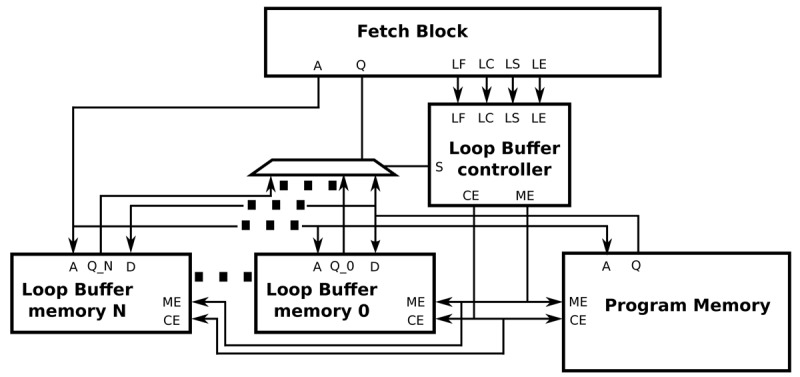
IMO interface for a BCLB architecture.

Figure 10 shows how the BCLB architecture is composed of different loop buffer memories. In a BCLB architecture, every memory is connected to the processor architecture and the program memory through multiplexers. The loop buffer controller, based on the loop body size of the loop that is on execution, decides which of the available loop buffer memories is connected directly with the program memory and the processor. The logic circuit that decides if the loop buffer architecture is activated is the same as the one used in the SCLB architecture. In order to make all the decisions that are described previously, the complexity of the state-machine is incremented. However, Figure 10 shows that this modification allows the design of the loop buffer architecture to be scalable.

## 5. Experimental Evaluation

This section shows the results of the experimental evaluation of the SCLB and the BCLB architecture. Firstly, Subsection 5.1 describes the methodology that is used in our energy simulations. Secondly, Subsection 5.2 analyses the experimental applications that are described in Section 3 based on profiling information. Finally, Subsection 5.3 shows and discusses the results of the power simulations.

### 5.1. Simulation Methodology

The simulation methodology that is used in our experimental evaluation is described by the following steps:

**Application mapping** The selected application is mapped to the system architecture that we want to simulate. The I/O data connections of the system are used by the embedded systems designer to corroborate the correct functionality of the system.
**Behaviour simulation** The mapped application is simulated on the system architecture in order to check its correct functionality. For that purpose, an Instruction-Set Simulator (ISS) from Target Compiler Technologies [29] is used.
**RTL implementation** The RTL language description files of the processor are automatically generated using the HDL generation tool from Target Compiler Technologies [29]. The design of the interfaces between the DMH and the IMO has to be added in order to have a complete description of the whole system in RTL language.
**RTL synthesis** When every component of the system has its own RTL language description file, the design is synthesised. In our RTL synthesis, a 90 nm Low Power TSMC library is used for a system frequency of 100 MHz. During synthesis, clock gating is used whenever possible.
**Place and route** After the synthesis, place and route is performed using Encounter [32].
**Recording Activity** It is necessary to generate a Value Change Dump (VCD) file for the desired time interval of the netlist simulation. If the selected time interval is the execution time of the application, the VCD file will contain the information of the activity of every net and every component of the whole system when an input data frame is processed.
**Extraction of power consumption** As a final step, the information of the average power consumption is extracted with the help of Primetime [33].

**Figure 11.**
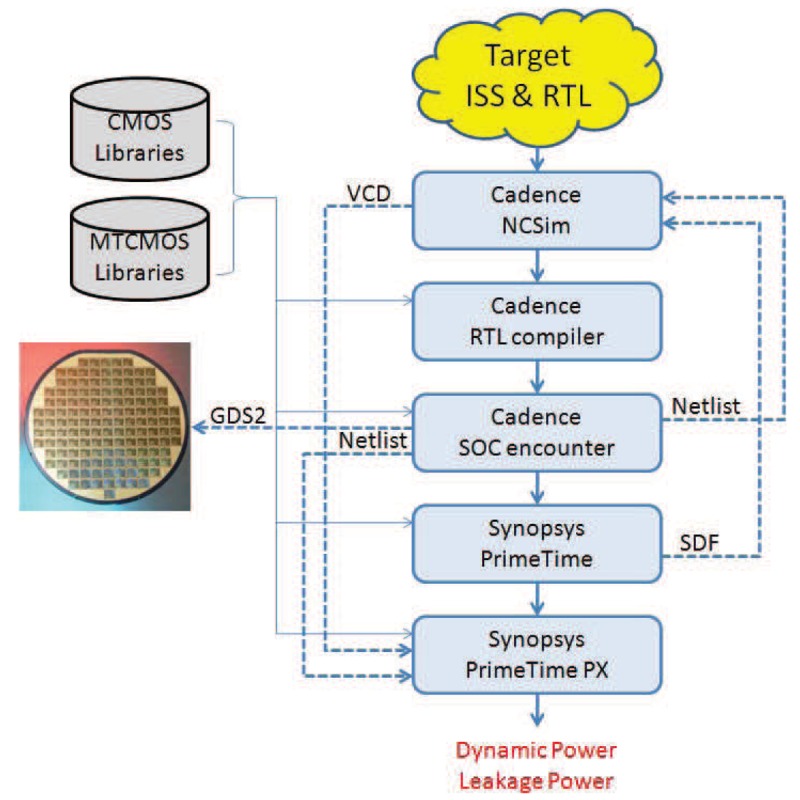
Simulation Methodology.

Figure 11 shows the inputs and outcomes of each step described above.

### 5.2. Analysis of the Experimental Applications

**Figure 12.**
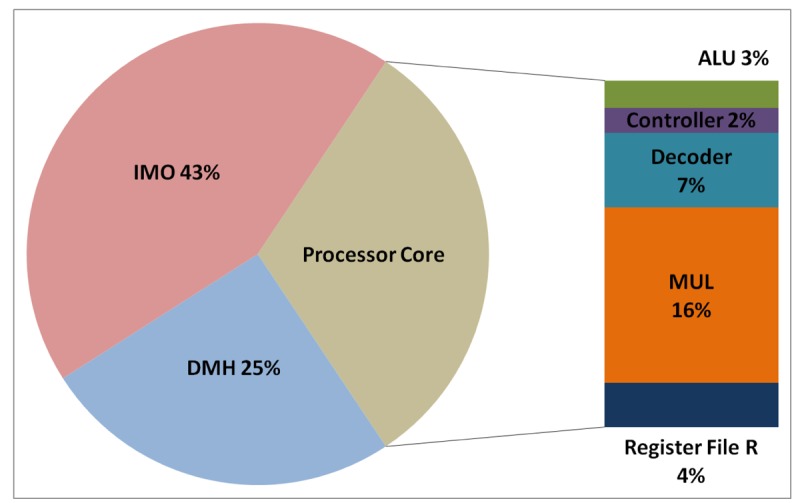
Power breakdown in the general-purpose processor running the HBD algorithm.

**Figure 13.**
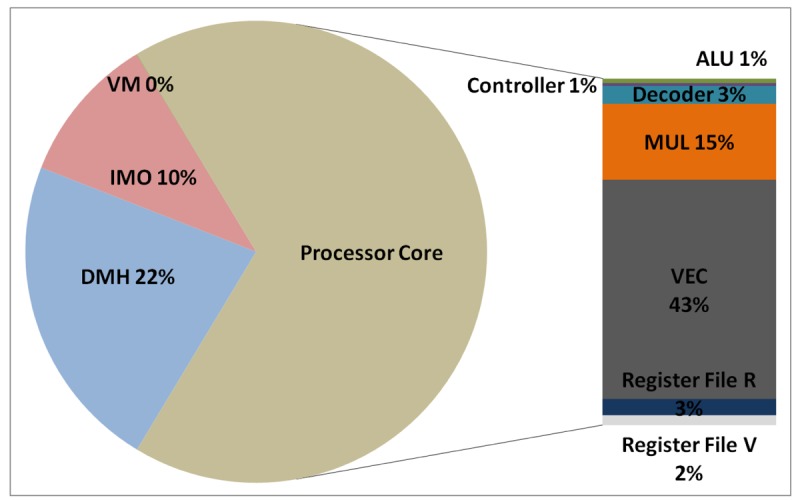
Power breakdown in the optimised processor running the HBD algorithm.

**Figure 14.**
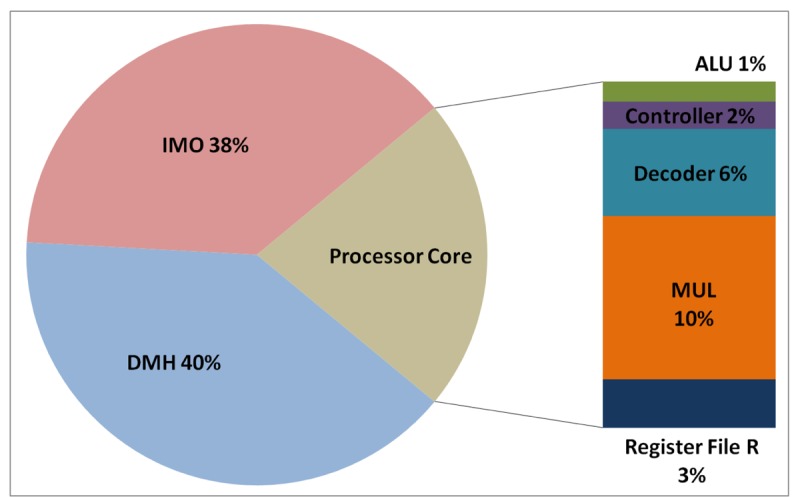
Power breakdown in the general-purpose processor running the AES algorithm.

**Figure 15.**
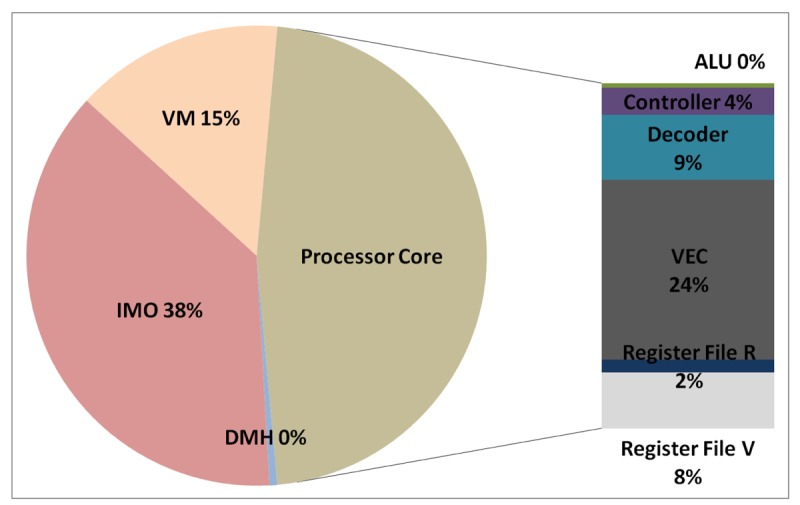
Power breakdown in the optimised processor running the AES algorithm.

The total energy consumption of the systems that are presented in this paper is strongly influenced by the consumption of the IMO. Following the steps that are described in Subsection 5.1, Figures 12, 13, 14, and 15 present the first outcome from the experimental evaluation. Figures 12 and 13 show the power breakdowns that are related with the HBD algorithm, whereas Figures 14 and 15 show the power breakdowns that are related with the AES algorithm. In these figures, the components of the processor core are grouped. Apart from seeing how the power distribution changes from a design based on a general-purpose processor to an ASIP design, these figures demonstrate that the total energy consumption of these systems is strongly influenced by the consumption of the IMO.

**Figure 16.**
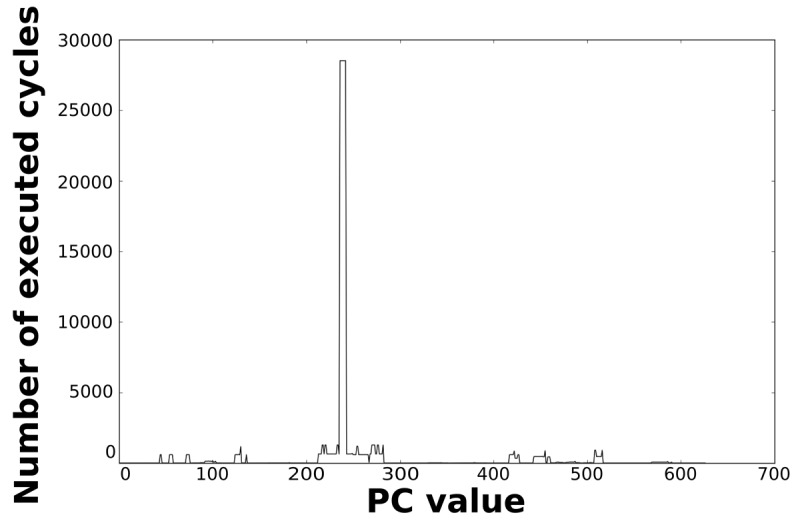
Number of cycles per PC in the general-purpose processor running the HBD algorithm.

**Figure 17.**
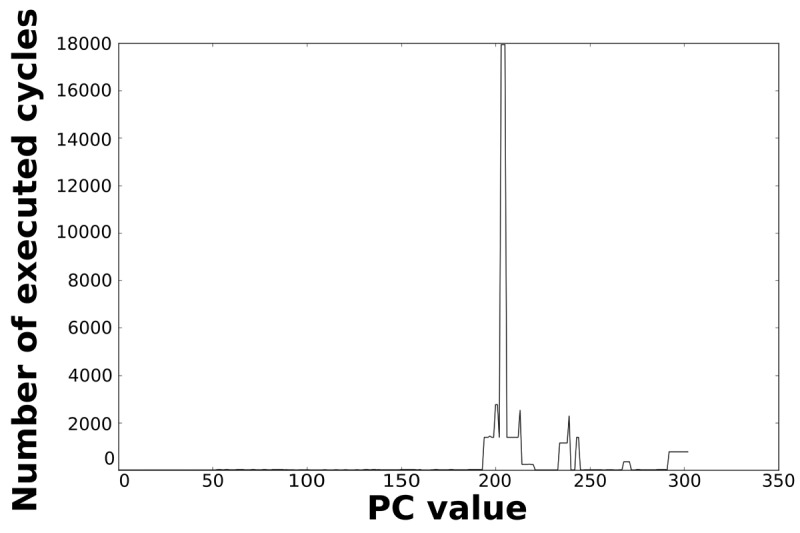
Number of cycles per PC in the optimised processor running the HBD algorithm.

**Figure 18.**
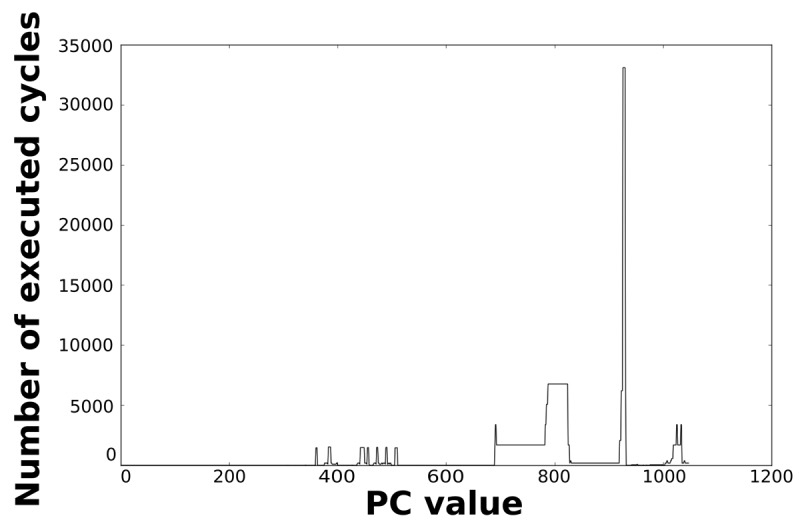
Number of cycles per PC in the general-purpose processor running the AES algorithm.

**Figure 19.**
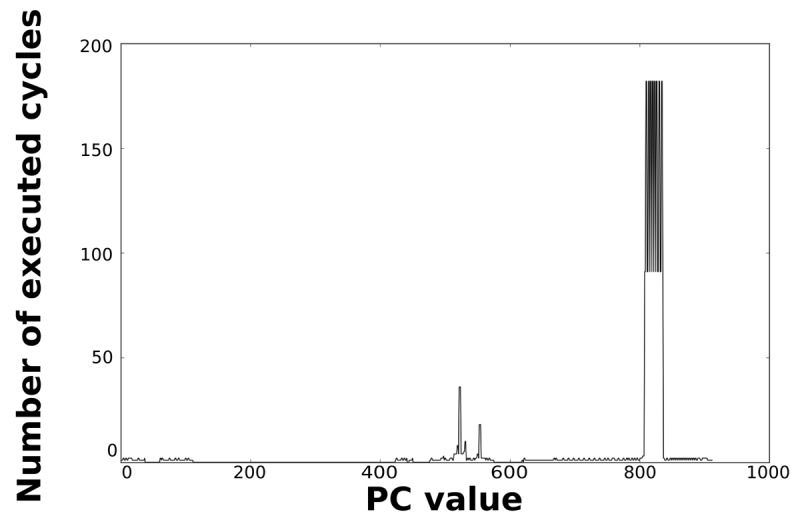
Number of cycles per PC in the optimised processor running the AES algorithm.

Loops dominate the total energy consumption of the IMO. Figures 16, 17, 18, and 19 show profiling information based on the accesses that are done in the program address space. Figures 16 and 17 show the profiles based on the number of cycles per program counter that are related with the HBD algorithm, whereas Figures 18 and 19 show the profiles based on the number of cycles per program counter that are related with the AES algorithm. We can see from these Figures that there are regions that are more frequently accessed than others. This situation implies the existence of loops. Apart from this fact, it is possible to see from these figures that the application execution time of the selected applications is dominated by only a few loops.

In order to implement energy efficient IMOs based on loop buffer architectures, more detail information related with loops is needed. Tables 1, 2, 3, and 4 provide this information. Tables 1 and 2 present the loop profiling information of the systems that are related with the HBD algorithm, whereas Tables 3 and 4 present the loop profiling information of the systems that are related with the AES algorithm. In these tables, loops are numbered in the static order that they appear in the assembly code of the algorithm. A nested loop creates another level of numbering. Thus, a loop named *2* corresponds to the second loop encountered, while a loop named *2.1* corresponds to the first sub-loop encountered in the loop named *2*. These tables corroborate the fact that the execution time of the loops dominates the total execution time of the application. For instance, the execution time of the loops represents approximately 79% of the total execution time of the HBD algorithm in the case of the general-purpose processor, and 81% in the processor that is optimised for this algorithm. In contrast, in the AES algorithm, the execution time of the loops represents 77% of the total execution time in the case of the general-purpose processor, and 90% in the processor that is optimised for this algorithm. It is necessary to remark that differences exist between algorithms of the same application due to the source code transformations and mapping optimisations that are applied in the optimised algorithms in order to generate efficient codes.

The configurations of the SCLB and BCLB architectures that are analysed in this paper are based on the loop profiling presented in Tables 1, 2, 3, and 4. On the one hand, the selection of the SCLB configurations is based on the small size of the loops that have bigger percentage of execution time. With this strategy, we assume that these configurations are the most energy efficient. This assumption is based on the fact that these configurations provide the highest energy savings among all the possible configurations. These major energy savings help to reduce the penalty related with the introduction of the loop buffer architecture in the system. On the other hand, the selection of the BCLB configurations is based on the strategy of taking the maximum loop body size of the application, and chop it by the granularity of the smaller loop body size that the applications contains. This strategy is used in these architectures, because the exact energy consumption of the extra logic that has to be added in the loop buffer controller is unknown. Table 5 presents the initial configurations that are evaluated.

In order to conclude the analysis of the experimental applications, it is necessary to remark that due to time requirements, a system frequency of 100 MHz is fixed. At this frequency, the HBD algorithm running on the general-purpose processor spends 462 cycles in order to process an input sample contained in the data frame. However, if this algorithm is running on the processor that is optimised for this algorithm, the number of cycles in order to process an input sample contained in the data frame is 11 cycles. On the other hand, the AES algorithm running on the general-purpose processor spends 484 cycles in order to process an input sample contained in the data frame. If this algorithm is running on the processor that is optimised for this algorithm, the number of cycles in order to process an input sample contained in the data frame is only 3 cycles.

### 5.3. Power Analysis

Tables 6, 7, and 8 present the power results for each system that is evaluated. These tables show the dynamic power, the leakage power, and the total power for all the configurations that are presented in Table 5. As it can be seen, the power consumption of the IMO is the sum of the power that is consumed by the components that the IMO contains (*i.e.*, the loop buffer controller, the loop buffer memory and the program memory).

We can see from these tables that the systems that are optimised for the experimental applications always consume less power than the general-purpose systems. Therefore, the introduction of the SCLB and BCLB architectures does not affect this energy consumption trend.

Analysing Table 7, it is possible to see that there is a decrease on the dynamic power of these systems in relation with the baseline architectures. This is because the majority of the instructions are fetched from a small memory instead of the large memory that forms the program memory. On the other hand, the SCLB architectures have an increase in the leakage power consumption in relation with the baseline architectures, due to the introduction of the loop buffer architecture. We can see also the importance of the loop buffer controller in the IMO, which accounts from the 5% of the power consumption of the IMO in the system where the AES algorithm is running on the general-purpose processor, to 30% in the system where the AES algorithm is running on the processor that is optimised for this algorithm.

Using the profiling information presented in Tables 1, 2, 3, and 4, and the power results obtained from the simulations of the systems presented in Table 5, we can evaluate if our initial configurations for the SCLB architecture are selected correctly from the energy consumption point of view.

**Figure 20.**
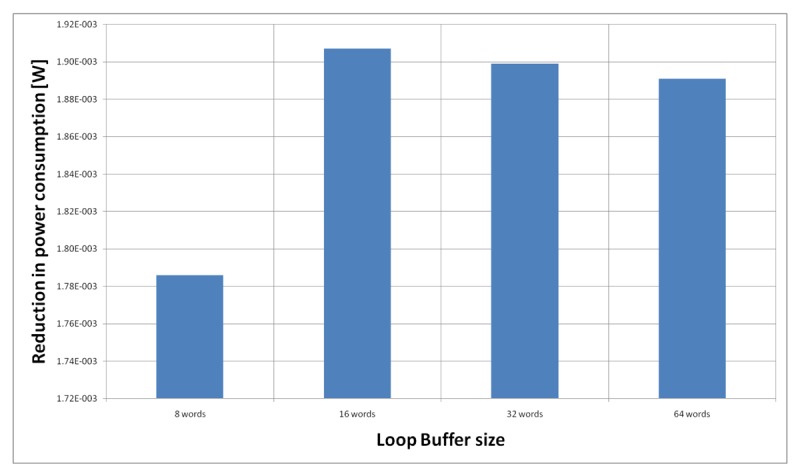
HBD algorithm running on the general-purpose processor using different configurations for the SCLB architecture.

For the HBD algorithm running on the general-purpose processor, Figure 20 shows the power reductions that we can achieve for all the possible configurations. In the configuration of eight words, the 73% of the execution time of the application is on loops, while in the rest of the configurations this percentage is 79%. We can see that in this scenario, the best configuration is a loop buffer memory of 16 words, because the increase of use of the loop buffer memory compensates the penalty introduced by using a bigger loop buffer architecture.

**Figure 21.**
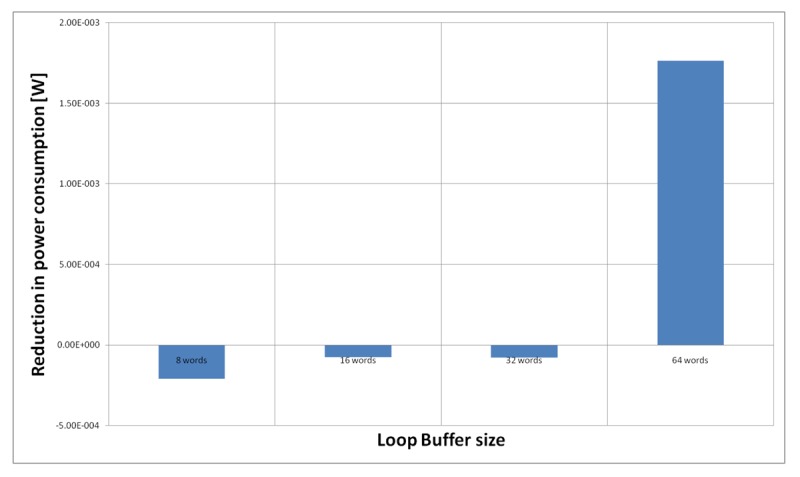
HBD algorithm running on the optimised processor using different configurations for the SCLB architecture.

Figure 21 shows the energy reductions we can achieve for all the possible configurations when the HBD algorithm is running on the processor that is optimised for this algorithm. In the configuration of eight words, the 2% of the execution time of the application is on loops. This percentage is 11% in the configuration of 16 and 32 words, whereas in the configuration of 64 words this percentage is 81%. We can see that in this scenario, the only configuration that brings energy savings is the loop buffer of 64 words. The percentages of the execution time of the rest of configurations do not compensate the penalty introduced by using a loop buffer architecture.

**Figure 22.**
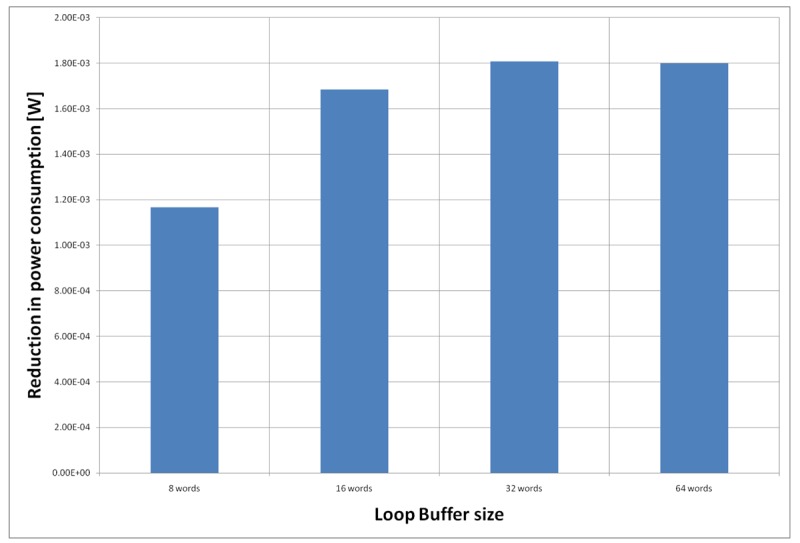
AES algorithm running on the general-purpose processor using different configurations for the SCLB architecture.

For the AES algorithm running on the general-purpose processor, Figure 22 shows the energy reductions we can achieve for all the possible configurations. In the configuration of eight words, the 47% of the execution time of this application is on loops; in the configuration of 16 words this percentage is 70%; in the configuration of 32 words this percentage is 75%, whereas in the configuration of 64 words this percentage is 77%. We can see that in this scenario, the best configuration is a loop buffer of 32 words, because the increase of use of the loop buffer architecture compensates the penalty introduced by using a bigger loop buffer memory. On the other hand, the small increase in the percentage of execution time from the configuration of 32 words to 64 words does not compensate the increase in leakage consumption that this last loop buffer architecture has.

**Figure 23.**
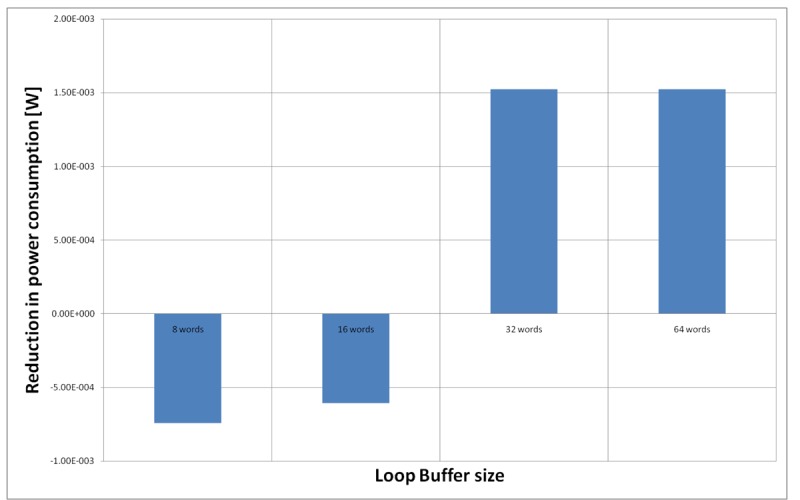
AES algorithm running on the optimised processor using different configurations for the SCLB architecture.

Figure 23 shows the energy reductions we can achieve for all the possible configurations when the AES algorithm is running on the processor that is optimised for this algorithm. In the configuration of 8 words, the 5% of the execution time of the application is on loops; in the configuration of 16 words this percentage is 6%, whereas in the configuration of 32 and 64 words this percentage 90%. We can see that in this scenario, the best configuration is a loop buffer of 32 words. The percentages of execution time for the 8 and 16 words configurations do not compensate the penalty introduced by using a loop buffer architecture. Also in this scenario, the small increase in the execution time percentage from the configuration of 32 words to 64 words does not compensate the increase in leakage power consumption that this last loop buffer architecture has.

Analysing Table 8, it is possible to see that also in these architectures, there is a decrease in the dynamic power of these systems in relation with the baseline architectures. However, we can see that these architectures sometimes do not offer as good energy savings as the SCLB architectures offer, because the system suffers an increase in both dynamic and leakage power consumption with the introduction of these loop buffer architectures. Firstly, in the dynamic power consumption, the loop buffer controller of the BCLB architecture has higher complexity than in the SCLB architecture. Secondly, in the leakage power consumption, apart from the higher complexity of the loop buffer controller, there is more loop buffer memories. In these architectures, the importance of the loop buffer controller is increased in the IMO, which now accounts for 10% of the power consumption of the IMO in the AES algorithm when it is running on the general-purpose, and for 32% in the HDB algorithm running on the processor that is optimised for this algorithm. Using the same information and methodology as in the analysis of the SCLB architectures, we can analyse if our configurations for the BCLB architectures are power efficient.

**Figure 24.**
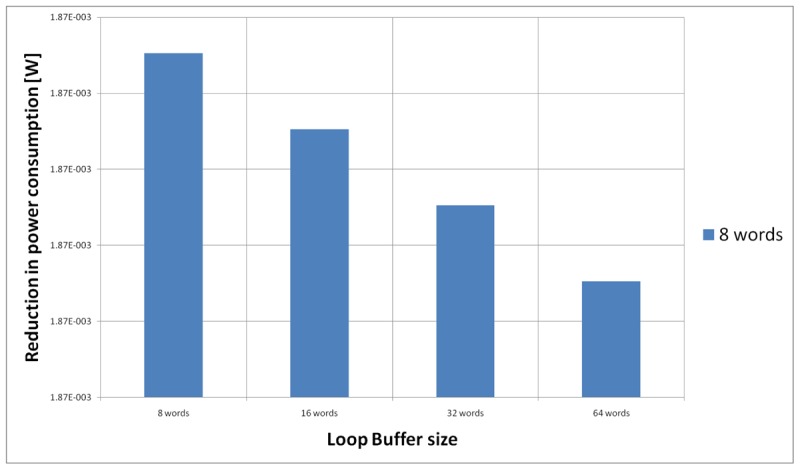
HBD algorithm running on the general-purpose processor using different configurations for the BCLB architecture.

For the HBD algorithm running on the general-purpose processor, we have to analyse only the loop buffer configurations of 8 instruction words, because all the loops can fit in a loop buffer of 16 instructions words (see Table 1), and every configuration in a BCLB architecture with a loop buffer of 16 instruction words is worse in power consumption than a SCLB architecture of 16 instructions words. Figure 24 shows the possible configurations of two loop buffers, where one of them has a fixed size of 8 words. From this Figure, we can see that the best configuration is two loop buffers of 8 words. If we compare the energy savings from the BCLB and the SCLB architecture, we can see that for this specific scenario, it is better to have the SCLB architecture.

**Figure 25.**
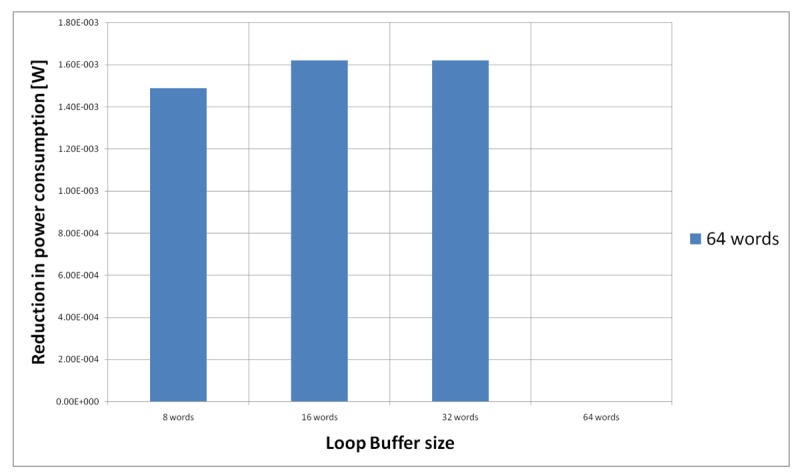
HBD algorithm running on the optimised processor using different configurations for the BCLB architecture.

For the HBD algorithm running on the processor that is optimised for this algorithm, we have to analyse only the loop buffer configurations of 64 instruction words because any configuration without a loop buffer of this size will not bring us energy savings (see Figure 21). Figure 25 shows the configuration of two loop buffers, where one of them has a fixed size of 64 words. From this Figure, we can see that the best configuration is a loop buffer of 16 words together with the loop buffer of 64 words. If we compare the energy savings from the BCLB and the SCLB architecture, we can see that for this specific scenario it is also better to have the SCLB architecture.

**Figure 26.**
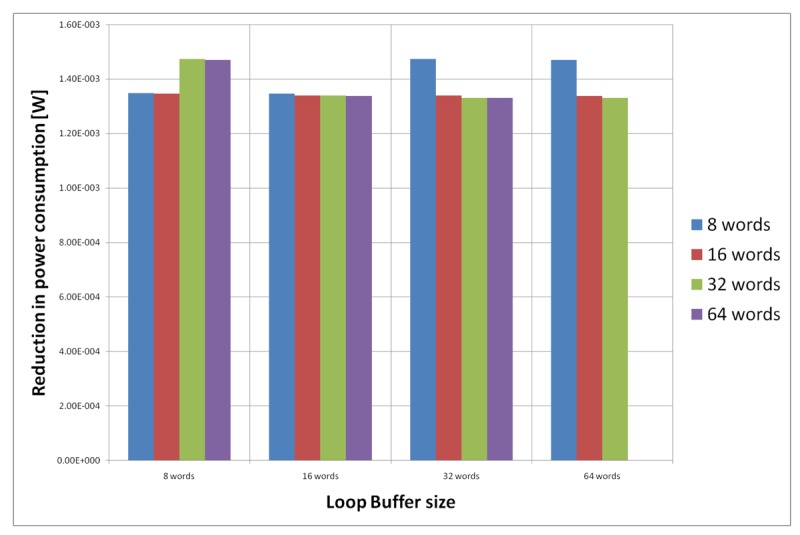
AES algorithm running on the general-purpose processor using different configurations for the BCLB architecture.

For the AES algorithm running on the general-purpose processor, we have to analyse all the possible configurations because the execution time of the application is spread (see Table 3). The configuration with two loop buffers of 64 instruction words each is not analysed, because this configuration is worse in energy efficiency than the SCLB architecture of 64 instructions words, due to the increase in energy consumption of the loop buffer controller. From Figure 26, we can see that the best configuration is a loop buffer of 8 words together with a loop buffer of 32 words. In this case, if we compare the energy savings from the BCLB and the SCLB architecture, we can see that for this specific scenario it is also better to have the SCLB architecture.

**Figure 27.**
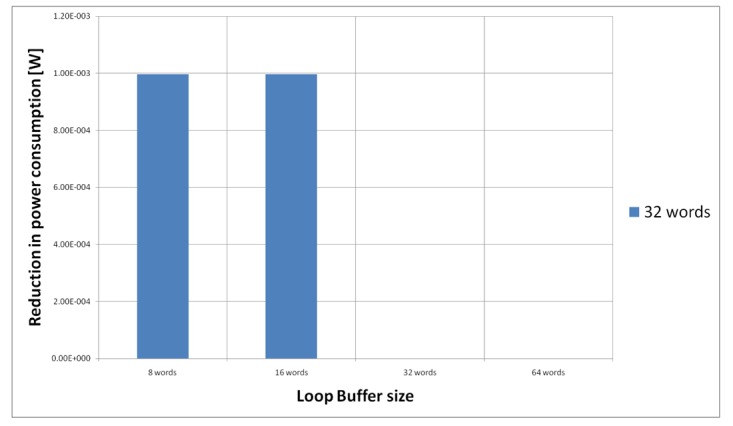
AES algorithm running on the optimised processor using different configurations for the BCLB architecture.

For the AES algorithm running on the processor that is optimised for this algorithm, we have to analyse only the loop buffer configurations that has 32 instruction words, because all the loops can fit in a loop buffer of 32 instructions words (see Table 4). However, from Figure 23, we can see that only loop buffers of 32 and 64 instruction words bring us energy savings. Therefore, we will analyse only the loop buffer configurations that has 32 instructions words. Figure 27 shows the configuration of two loop buffers, where one of them has a fixed size of 32 words. From this figure, we can see that the best configuration is a loop buffer of 8 words together with the loop buffer of 32 words. If we compare the energy savings from the BCLB and the SCLB architecture, we can see that for this specific scenario it is also better to have the SCLB architecture.

**Figure 28.**
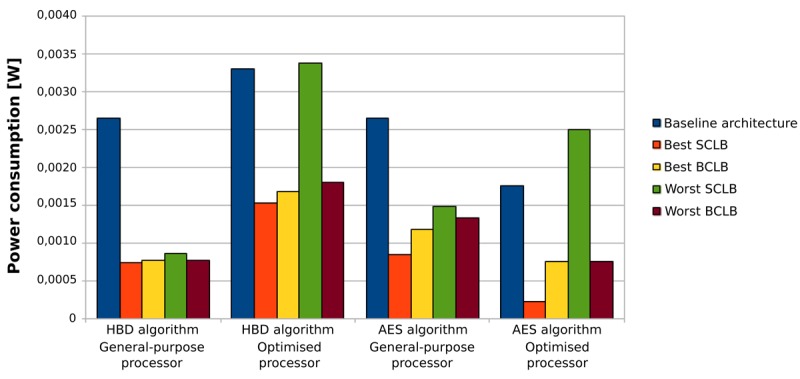
Summary of the best and worst SCLB and BCLB architectures.

Based on all the previous results and discussions, we can conclude that the use of loop buffer architectures in order to optimise the IMO from the energy efficiency point of view should be evaluated carefully. In the case studies that are presented in this paper, the SCLB architecture is normally more energy efficient than the BCLB architecture, as can be seen in Figure 28. However, the SCLB architecture is not always more energy efficient than the BCLB architecture. The higher energy efficiency of the SCLB architecture is because the whole execution time of all benchmarks is concentrated in a few loops with similar loop body size. If we can find a benchmark where this percentage is shared between loops with different loop body sizes, the BCLB architecture will then bring us more energy efficiency than the SCLB architecture. Therefore, the two factors to take in account in order to implement an energy efficient IMO based on a loop buffer architecture are:

- the percentage of the execution time of the application that is related to the execution of the loops included in the application. If this percentage is low, the introduction of a loop buffer architecture in the IMO cannot offer any energy savings, because the loop buffer architecture is not used enough to achieve energy savings. In contrast, the higher this percentage, the higher energy savings that can be achieved.
- the distribution of the execution time percentage, which is related to the execution of the loops, over each one of the loops that forms the application. For instance, the whole execution time percentage that is related to loops can belong only to a few loops, or in another case, this percentage can be spread in each loop homogeneously. If the whole execution time is concentrated in a few loops, the SCLB architecture will bring more energy savings than the BCLB. If this percentage is distributed homogeneously between loops, the BCLB architecture will then bring more energy savings than the SCLB. These facts are based on the efficient use of the multi-banks that can form the loop buffer architecture.

## 6. Conclusions

In this paper, the loop buffer concept was applied in two real-life embedded applications that are widely used in biomedical WSNs. The loop buffer architectural organisations that were analysed in this paper were the Single Central Loop Buffer and the Banked Central Loop Buffer architecture. An analysis of the experimental applications that were used in this paper was performed to show which type of loop buffer scheme was more suitable for applications with certain behaviour. To evaluate the power impact, a post-layout simulation was used to have an accurate estimation of parasitics and switching activity. The evaluation was performed using TSMC 90 nm Low Power library and commercial memories. From the experimental evaluation, gate-level simulations demonstrated that a trade-off exists between the complexity of the loop buffer architecture and the power benefits of utilising it. This confirms our results, showing that the Central Banked Loop Buffer does not always bring benefits. Therefore, the use of loop buffer architectures in order to optimise the IMO from the energy efficiency point of view should be evaluated carefully. Two factors have to be taken into account in order to implement an energy efficient IMO based on a loop buffer architecture: (1) the percentage of the execution time of the application that is related with the execution of the loops included in the application, and (2) the distribution of the execution time percentage, which is related with the execution of the loops, over each one of the loops that forms the application.

## Figures and Tables

**Table 1.** Loop profiling of the HBD algorithm on the general-purpose processor.

	**Start address**	**End address**	**Loop body size**	**Number of iterations**	**Execution time [%]**
**Loop 1**	33	34	2	4	0
**Loop 2**	44	45	2	594	0
**Loop 3**	54	57	4	594	1
**Loop 4**	72	75	4	594	1
**Loop 5**	92	103	12	132	1
**Loop 6**	124	136	13	594	3
**Loop 7**	160	160	1	15	0
**Loop 8**	236	242	7	32,625	71
**Loop 9**	417	427	11	594	2
**Loop 10**	569	590	22	64	0

**Table 2.** Loop profiling of the HBD algorithm on the optimised processor.

	**Start address**	**End address**	**Loop body size**	**Number of iterations**	**Execution time [%]**
**Loop 1**	192	244	53	1,380	70
**Loop 1.1**	200	205	6	1	0
**Loop 2**	266	271	6	350	2
**Loop 3**	209	302	13	768	9

**Table 3.** Loop profiling of the AES algorithm on the general-purpose processor.

	**Start address**	**End address**	**Loop body size**	**Number of iterations**	**Execution time [%]**
**Loop 1**	307	309	3	8	0
**Loop 2**	324	327	4	2	0
**Loop 3**	340	342	3	16	0
**Loop 4**	360	362	3	1,460	3
**Loop 5**	383	387	5	1,600	7
**Loop 6**	409	411	3	4	0
**Loop 7**	419	421	3	8	0
**Loop 8**	426	428	3	16	0
**Loop 9**	436	458	23	92	2
**Loop 10**	472	474	3	1,392	3
**Loop 11**	489	491	3	1,392	3
**Loop 12**	506	510	5	1,460	6
**Loop 13**	519	523	5	4	0
**Loop 14**	926	930	5	6,016	25
**Loop 15**	942	1,000	59	40	2
**Loop 16**	1,019	1,034	16	1,692	26

**Table 4.** Loop profiling of the AES algorithm on the optimised processor.

	**Start address**	**End address**	**Loop body size**	**Number of iterations**	**Execution time [%]**
**Loop 1**	519	524	6	36	5
**Loop 2**	544	560	17	2	1
**Loop 2.1**	550	555	6	0	0
**Loop 3**	806	837	32	91	84

**Table 5.** Configurations of the experimental framework.

	**Baseline architecture**	**SCLB**	**BCLB**
**HBD algorithm**	No loop buffer	8 words	8 banks of
**General-purpose processor**	architecture		8 words

**HBD algorithm**	No loop buffer	64 words	8 banks of
**Optimised processor**	architecture		8 words

**AES algorithm**	No loop buffer	8 words	4 banks of
**General-purpose processor**	architecture		8 words

**AES algorithm**	No loop buffer	32 words	4 banks of
**Optimised processor**	architecture		8 words

**Table 6.** Power consumption [W] of the baseline architecture.

	**Component**	**Dynamic power**	**Leakage power**	**Total power**
**HBD algorithm**	IMO	4.44 × 10^−6^	0.91 × 10^−9^	4.44 × 10^−6^
**-**	LB Controller	0	0	0
**General-purpose**	LB Memory	0	0	0
**processor**	PM	4.44 × 10^−6^	0.91 × 10^−9^	4.44 × 10^−6^

**HBD algorithm**	IMO	3.57 × 10^−7^	8.46 × 10^−11^	3.57 × 10^−7^
**-**	LB Controller	0	0	0
**Optimised**	LB Memory	0	0	0
**processor**	PM	3.57 × 10^−7^	8.46 × 10^−11^	3.57 × 10^−7^

**AES algorithm**	IMO	1.81 × 10^−6^	4.32 × 10^−10^	1.82 × 10^−6^
**-**	LB Controller	0	0	0
**General-purpose**	LB Memory	0	0	0
**processor**	PM	1.81 × 10^−6^	4.32 × 10^−10^	1.82 × 10^−6^

**AES algorithm**	IMO	1.20 × 10^−6^	2.11 × 10^−10^	1.20 × 10^−6^
**-**	LB Controller	0	0	0
**Optimised**	LB Memory	0	0	0
**processor**	PM	1.20 × 10^−6^	2.11 × 10^−10^	1.20 × 10^−6^

**Table 7.** Power consumption [W] of the IMO based on an SCLB architecture.

	**Component**	**Dynamic power**	**Leakage power**	**Total power**
**HBD algorithm**	IMO	1.74 × 10^−6^	1.14 × 10^−9^	1.74 × 10^−6^
**-**	LB Controller	2.55 × 10^−7^	1.60 × 10^−10^	2.55 × 10^−7^
**General-purpose**	LB Memory	6.97 × 10^−8^	6.60 × 10^−11^	6.97 × 10^−8^
**processor**	PM	1.41 × 10^−6^	9.16 × 10^−10^	1.41 × 10^−6^

**HBD algorithm**	IMO	1.40 × 10^−7^	1.77 × 10^−10^	1.40 × 10^−7^
**-**	LB Controller	3.71 × 10^−8^	2.66 × 10^−11^	3.71 × 10^−8^
**Optimised**	LB Memory	5.76 × 10^−8^	6.56 × 10^−11^	5.76 × 10^−8^
**processor**	PM	4.50 × 10^−8^	8.46 × 10^−11^	4.51 × 10^−8^

**AES algorithm**	IMO	1.76 × 10^−6^	5.25 × 10^−10^	1.76 × 10^−6^
**-**	LB Controller	1.03 × 10^−7^	7.39 × 10^−11^	1.03 × 10^−7^
**General-purpose**	LB Memory	9.54 × 10^−9^	2.68 × 10^−11^	9.54 × 10^−9^
**processor**	PM	1.65 × 10^−6^	4.25 × 10^−10^	1.65 × 10^−6^

**AES algorithm**	IMO	8.32 × 10^−7^	4.12 × 10^−10^	8.36 × 10^−7^
**-**	LB Controller	2.43 × 10^−7^	7.53 × 10^−11^	2.47 × 10^−7^
**Optimised**	LB Memory	1.79 × 10^−7^	1.29 × 10^−10^	1.79 × 10^−7^
**processor**	PM	4.10 × 10^−7^	2.13 × 10^−10^	4.10 × 10^−7^

**Table 8.** Power consumption [W] of the IMO based on a BCLB architecture.

	**Component**	**Dynamic power**	**Leakage power**	**Total power**
**HBD algorithm**	IMO	1.97 × 10^−6^	1.47 × 10^−9^	1.97 × 10^−6^
**-**	LB Controller	4.72 × 10^−7^	3.95 × 10^−10^	4.72 × 10^−7^
**General-purpose**	LB Memory	8.73 × 10^−8^	1.59 × 10^−10^	8.73 × 10^−8^
**processor**	PM	1.41 × 10^−6^	9.16 × 10^−10^	1.41 × 10^−6^

**HBD algorithm**	IMO	1.64 × 10^−7^	3.83 × 10^−10^	1.65 × 10^−7^
**-**	LB Controller	5.51 × 10^−8^	1.40 × 10^−10^	5.51 × 10^−8^
**Optimised**	LB Memory	6.39 × 10^−8^	1.58 × 10^−10^	6.39 × 10^−8^
**processor**	PM	4.50 × 10^−8^	8.46 × 10^−11^	4.51 × 10^−8^

**AES algorithm**	IMO	1.90 × 10^−6^	7.40 × 10^−10^	1.90 × 10^−6^
**-**	LB Controller	2.35 × 10^−7^	2.72 × 10^−10^	2.35 × 10^−7^
**General-purpose**	LB Memory	1.46 × 10^−8^	4.29 × 10^−11^	1.46 × 10^−8^
**processor**	PM	1.65 × 10^−6^	4.25 × 10^−10^	1.65 × 10^−6^

**AES algorithm**	IMO	6.60 × 10^−7^	4.30 × 10^−10^	6.60 × 10^−7^
**-**	LB Controller	5.20 × 10^−8^	1.10 × 10^−11^	5.20 × 10^−8^
**Optimised**	LB Memory	1.98 × 10^−7^	2.06 × 10^−10^	1.98 × 10^−7^
**processor**	PM	4.10 × 10^−7^	2.13 × 10^−10^	4.10 × 10^−7^
